# First Principle of Surgical Operation

**Published:** 1808-06

**Authors:** Alexander Walker

**Affiliations:** Lecturer on Physiology at Edinburgh


### Medical and Phyfical Journal.

VOL. XIX.] JUNE, 1808. [NO. 112.

*Printed for R. PHILLIPS, by W. Thorne, Red Lion Court, Fleet Street, London*

### FIRST PRINCIPLE OF SURGICAL OPERATION,

By ALEXANDER WALKER, Esq.

LECTURER ON PHYSIOLOGY AT EDINBURGH.

### DIVISION I.

*Of the vague, cruel, and unsuccessful Plans of Operation at present practised.*

THE vague plans of surgical operation at present practised, are too generally known and too sincerely deplored by every man of science and of feeling to demand much of comment on the present occasion.

How often, in the attempt to take up an artery, does the ignorant and *timid* operator make an incision so limited that he is afterwards forced cruelly to extend it? How often, having extended his incision, does he find no artery beneath it, and dig or mine for it in a new direction by cutting up the flesh of the patient? How often, having reached the place he sought for, does he mistake another artery, a nerve, or even a vein, for that which ought to be the subject of operation? How often, having found the artery, is he at a loss to recognise it? What trepidations and alarms does not this operator, more miserable even than the patient himself, endure?

I know no situation more wretched or more pitiable than this. Here, even at the best, the most exquisite torture is inflicted; the poor trembling patient, abstracted from every other earthly object, and with the most exquisite feelings concentrated on the present, awaits in awful suspense the impending stroke which is to divide the skin, that seat of accumulated sensibility, and deliver him from wretchedness; but the hope is vain; his torments are again and again renewed; he perceives that his life is the sport of a fool; he writhes in agony or sinks in deliquium, and the issue is, as might be expected, too generally, even at the best, unsuccessful.

When all this is not the case, how often does the operator, on the contrary ignorant and *rash*, plunge his knife into some vital organ of the patient, and at once terminate his life and his diseases?

That this picture is not too highly coloured, I solemnly appeal to the consciences and the feelings even of the best operators, who are too candid to deny, that melancholy reflection has often oppressed them from the fate of patients—martyrs to their want of precision.

### Division II.

### *Of the consequent Importance of this Inquiry.*

To obviate this train of miseries; to give ease and satisfaction to the operator, by conferring precision and success upon his operation, and to rescue from death, or rather from torture and agony, innumerable sufferers, are the results which I confidently expect from the adoption of the following method. Nor can I say more in proof of its importance. I doubt not, that it will ultimately constitute the basis and direct the genius of surgery.

### Division III.

### *Enunciation of the Principle, and Observations respecting it.*

There exists no other method of effecting this purpose than the following: namely, that *there is, especially in such parts as are not liable to be deranged by voluntary motion, an invariable relation between the arrangements of internal and the situation of external parts which may justly be considered as accurate signs of them; in consequence of which, by adopting on each side of the nerve, artery, or other subject of operation, a fixed point, or one rendered fixed by particular positions of the body, and by dividing, by a line or rule, the space between these fixed points into a given number of proportional parts, i. s. parts not equally large in all subjects, but uniformly of the same size with the relation to the whole which they compose, and generally differing in the male and female—by these means, I say, the situation of any given subject of operation, in any given part of the body, may be most accurately indicated, as being undeviatingly so many proportional parts distant from the one and so many from the other of these points; and thus does Anatomy, by what I term precise or real proportional measurements between fixed points, afford an obvious and simple, though hitherto undiscovered,*

*covered, general means of rendering the operations of Surgery perfectly precise and easy.* Indeed, such wonderful precision and ease do these real proportional measurements confer, that, by means of them, the finest needle may be passed from the surface of the body, through any great nerve or vessel that is liable to be the subject of Surgical operation.

Having stated this principle, I may merely observe, that, proceeding with all the ease and security which accuracy must infallibly confer, it is, in all operations, the first duty of the operator to ascertain by feeling, as nearly as possible, the thickness of adipose substance situated immediately under the skin of the patient, and then, with a bold and steady hand, to make an incision, deep in proportion to the thickness of that substance, and long in proportion to its depth, as well as to the additional depth and extent of the part to be operated upon.

These general principles excepted, other subordinate rules are chiefly applicable to particular operations.

### Division IV.

### *An Example of its Application.*

In order to illustrate this general principle, I cannot adduce a fairer or a better example than that which, according to it, is necessary to direct the taking up the subclavian artery above the clavicle.

In expressing positions connected with the following real measurement, I shall (as few gentlemen are acqnainted with them) use the accurate terms of Dr. Barclay, by whom Anatomy has been rescued from a barbarous and contemptible language, and to whom it owes the only one in which it can be at once easily and accurately understood or communicated.*

In consequence of having made use of it, I must also mention here what I consider a little improvement upon that system, which may be useful to those who are well versed in the combination of its terms, and are desirous of the utmost precision in anatomical description, and which may be illustrated by a single example; thus I should propose, that a line drawn along the middle of the atlantal side of the clavicle should be termed its atlantal line;

* As one proof of the imperfection of the previons language of anatomy, I may just mention, that Albinus and Soemmerring, for example, not only contradict each other, but that each frequently contradicts himself, in the use of the common terms *in priora, in exteriora, &c.*

that a line drawn along the middle of its central side should be termed its central line ; that a line equi-distant from these, should be termed its central and atlantal or its atlantal and central line ; that a line equally distant from its atlantal, and central and atlantal line, should be termed its atlanto-central ; and that a line equally distant from its central, and atlantal and central line, should be termed its centro-atlantal line. Thus, that beautiful system would, if necessary, be applicable to the most minute description.

In this operation, the patient ought to rest on the fundament—his body slightly inclining backward, so as to form an obtuse angle with the thighs — his neck and head inclined further backward, so as to form with the trunk an angle similar to that formed by it with the lower extremities, the inferior of these angles being concave, the other convex (this last indeed may more properly be called an irregular segment of a circle formed in consequence of the vertebræ of the neck bending in that direction.) In this position, therefore, the neck is in reality in the same parallel with the thighs. The head of the patient also is to be turned to the opposite side from that on which the operation is to be performed, where also the operator must stand.

In this case, then, the bringing of the shoulder sterno-sacrad, or forward and downward, which I deem indispenble, however necessary to the ease of the operator, is not very material to the correctness of the measurement. It is, however, of some consequence; for as the subsequent measurement is dependent on the clavicle, and as that bone describes a portion of a circle in having its lateral end brought sternad or toward the sternum, every single point of it would, in that position, have a different relation to the vessels beneath from that which each would have had if kept in its usual position.

Here then the shoulder being in this position, the head inclined backward and to the opposite side from that on which the operation is to be performed, and the arm held close by the side or in the atlanto-sacral direction, the measurement commences, and the fixed point chosen on each side of the immediate subject of operation are the two ends of the clavicle ; as nearly between these as can be done, consistently with keeping quite clear of the bone, a line must be drawn atlanto-central or upward and inward from it, and the given number of proportional parts into which this line must be divided are seven in number.—

number.—And now the situation of the artery may be most accurately indicated as being undeviatingly three of these proportional parts distant from the mesial or inner, and four from the lateral end of the clavicle; in consequence of which, if a fine needle, held at right angles to the clavicle, were directed centro-sacrad or inward and downward, it would, at 1 & ¼ of the above mentioned parts from the outside of the clavicle, reach the artery, and, being pushed farther, would pass through it.

This is an absolute demonstration of the precision and ease attending the principle I have announced, and of its applicability even to the most difficult of all operations.

In order, however, to perform the operation throughout, the point which I have indicated must be the centre of an incision through the integuments made in the line above mentioned, and must extend at least two parts mesiad or inward, and two laterad or outward, from the central point. Over the Trapezius laterad, and over the clavicular attachment of the sterno-cleido-mastoid muscle mesiad, it is not adviseable to cut deeper than the integuments, which sufficiently relieves the hand of the operator without the present execrable practice of dividing the last mentioned muscle; a practice not only unnecessary, but highly faulty, as this attachment serves as one of those particular points, above alluded to, (incidental to certain operations) which, by means of its edge, kept lax during the operation, very nearly directs the knife to a small protuberance on the atlanto-sternal or upper and outer surface of the first rib, to which the scalenus primus is attached, and immediately laterad or external to which the artery itself lays.

Above all, the incision ought to be cautiously continued mesiad or inward, in order to avoid the large veins situate there. Various branches of these veins may come in the way, and the more considerable may in general be easily avoided, but, if not, may easily be tied.—In continuing the incision centro-sacrad or inward and downward, an ascending branch of a vein is also in the way, and when considerable, it may, previous to the continuance of the incision, be tied. The incision, as you pass centro-sacrad or downward and inward, must be very limited indeed, as the jugular vein is internal and a twig of the brachial nerve external to it, both of which are much exposed to injury.—When the incision has been made one and one-fourth of the proportional parts sacro-centrad or downward and inward from the outside of the clavicle, the artery is

 reached reached. It lays external to that protuberance of the first rib, into which, as already observed, the scalenus primus is inserted, and consequently external to the insertion of the muscle itself.—The nerve, and particularly its branch, is latero-sternal or outward and forward from the artery, and before the operator attempts to take this up, he ought to have seen both of the former.—The course of the artery in descending is obliquely laterad or outward, and the precise space in which it lays resembles an isosceles triangle formed by the clavicle, clavicular portion of the sterno-cleido-mastoid and the omo-hyoideus; the clavicular portion of the sterno-cleido-mastoid being its base.

These directions are so precise, that, were the operator blind and the artery destitute of pulsation, he need not scruple to take up that which presents itself in the above mentioned situation.

By similar measurements, may every nerve, artery or vein in the body, liable to be the subject of surgical operation, be discovered with similar ease and precision, nor does there exist any other method of effecting this purpose.

### Division V.

*Objections to the Method proposed.*

All the objections to this method seem to me to be reducible to three kinds.

1st. It may be objected, that the clavicles, for example, may be fractured, and the fixed points deranged, and that, consequently, these accurate proportional measurements would be impracticable.

2d. It may be objected, that varieties in the distribution of arteries will render such precise measurements abortive.

3d. It may be objected, that disease often displaces arteries, and that there also precise measurements would be inapplicable.

### Division VI.

*Objections Answered.*

To these objections, I answer,

1st. That if the clavicle were fractured, still its mesial or sternal fixed point—its attachment to the sternum, or that to the scapula, would remain; and this being the case, it would only be necessary to take the measurement from the other clavicle, to examine how many absolute inches its three mesial or four lateral proportional parts make, and and to measure as many inches from the remaining fixed points of the fractured clavicle, which would exactly indicate the situation of the artery. There can only exist a single difficulty in this conversion of the proportional parts of one side into absolute ones on the other, viz. that the two clavicles may not be formed alike. This, however, does not take place to such an extent as to derange the operation in one case in a thousand; and it is scarcely to be expected, that any rule of this kind, however excellent, should possibly apply to that which might rather be called *comparative* than human anatomy.—Yet, even in this improbable case, every man of sense will grant, that the Surgeon who, upon the principle I have proposed, knows the precise situation in those which are regularly formed, will far more easily discover the monstrous one than he who knows neither; the former will make only one guess, the latter two, and, consequently, will have at least double the chance of failure.

2d. As to varieties of distribution, they take place only in certain situations, viz. where numerous muscles or movable parts surrounding them, produce deviation. This is not the case with the principal vessels of the head, neck, trunk, or upper parts of the extremities — the seats of almost all the great operations where arteries are to be discovered. It only happens in the middle and inferior parts of the extremities; and surely, even when varieties occur in such parts, the Surgeon who knows the precise situation in the great majority, will far more readily discover deviations than he who knows neither; the former will, I must again repeat, make one guess, the latter two, and, consequently, run at least double the risk of failure.

As to the displacement of arteries by disease, it can scarcely occur to any extent but from such diseases as produce evident external signs of that derangement; and here the precise Surgeon will be still less at a loss.

### Division VII.

### *A short History of the Method proposed.*

The want of some guide to the situation of internal parts has long been felt. Fixed points have been adopted, but with no regularity. Measurements, guessed by the eye, have sometimes been used, but even they have almost always been absolute, i. s. measurements from one point; rarely proportional, i. s. measurements between two points. The few guessed proportional measurements, however, as, for paracentesis abdominis, paracentesis thoracis,

&c. which have been mentioned in surgical works, have, it must be confessed, been such as, from the magnitude of the parts with which they were concerned, required little more than guess. Such, however, as they were, they evidently depended upon no principle, but seem as if hit upon accidentally, and, at all events, were never generally applied, nor ever gave origin to any general principle of surgical operation, except perhaps in the following instance.

It is a duty I owe to my friend Dr. Barclay to mention, that, in reading this paper to him, he observed, that he had been for some years in the habit of inculcating, *as a general principle,* these guessed proportional Measurements. I believe he is almost the only person who has even gone so far; and thus far the coincidence is valued by me. His measurements, however, were only guessed by the eye, as I have already said, and not real ones, which had never struck him or been practised by him.

Precise or real proportional measurements between fixed points, applicable to minute objects, i. s. such measurements as I have proposed and described, never have been, as far as I can discover, proposed and described before.

Even anatomists have been ignorant, that arteries (those liable to be deranged by voluntary motion excepted) are invariably situate at certain proportional distances from fixed points. I cannot prove this by a fairer or better example than the plates of Haller, *in which the same artery does not possess, in any two instances, the same relative situation.* And as these facts were unknown to anatomists, it is not to be wondered that surgeons were ignorant of them.

Now these precise or real proportional measurements between fixed points differ exactly as much from guessed ones, as the accurate cabinet, which was regulated in every part by the compass and square, would differ from the clumsy and unserviceable one, where every thing was constructed by guess. Here the parallel is so close, and the difference in the results so striking, that I could not have adduced a better illustration.

Impressed with its value, even Dr. Barclay permits me to say, that the precise or real proportional measurements proposed by me for ascertaining the situation of such internal parts as are not liable to be deranged by voluntary motion, viz. in the head, neck, and trunk, have his unqualified approbation.

The present proposition of precise or real proportional measuremeasurements between fixed points, was first mentioned by me, several years ago, to James Macartney, Esq. Lecturer on Comparative Anatomy and the Laws of Organic Existence, at St. Bartholomew's Hospital, and, in my opinion, one of the best comparative anatomists in Europe. I had no sooner announced it, than he assured me, that a similar method had struck himself; and I deem this, so far as authority goes, no mean testimony in behalf of its value.

I some time afterwards was preparing for the press, a work on the subject; but, in consequence ef his wishing to do so, and his very superior ability to execute such a work, I at that time abandoned it.

As Mr. Macartney performed upon the dead subject, in the Dissecting Room at St. Bartholomew's, the difficult operation I have just described, according to the direction I have given, and bestowed upon it also his warmest approbation, it is my duty to remark, that there is no person whose approbation could give me higher satisfaction.

Two or three years have now elapsed since this happened; and as the work of Mr. Macartney has not appeared, I trust I do not interfere with that gentleman's views in submitting to public consideration the principle which I have just enunciated.

### Division VIII.

### *The immense Danger arising from Ignorance of the previous Principle.*

I cannot better illustrate the danger arising from ignorance of the previous principle, than by quoting an instance of repeated failure in the very operation above described, in consequence of such ignorance.

The principle I have just mentioned was communicated to another Professor at an Hospital, not many miles distant from the last mentioned, generally reckoned one of the ablest Surgeons in London, who, possessing as much of illiberality as Mr. Macartney possessed of candour, sedulously avoided its adoption.

When illustrating the operation, therefore, in his Lectures, he first made the usual guessed cut, passing across and dividing one-half of the sterno-cleido-mastoid muscle, and, after some tearing and cutting, and innumerable complaints against the knives and the unfortunate assistant, told his pupils, that "they might think he had not yet got the artery, but he knew he had, and he should know it in the living subject, even if it did not pulsate." The subject, subject, however, was carried from Lecture, and the ligature was found to be firmly fixed upon the accompanying nerve.

This mistake was very properly communicated to the ingenious surgeon, who never forgave the discoverer of it; but very wisely planned how he might avoid the same mistake, or what he seemed to dread most—its consequences in the subsequent Course.

He had thus found, that ligatures left in the dead subject were more troublesome than in the living, and, having tied something which, to judge from the vague way in which he described it, might be a muscle as readily as an artery, he very properly, as his mind was not quite made up upon the subject, secured to all appearance his well-earned fame by cutting off and withdrawing the ligature, that no one might see what he had done. Unfortunately, however, curiosity and the wonder that it was possible for so eminent a surgeon to make so deplorable and dangerous an error, prompted a second examination of the subject. No ligature now remained to direct the Inquirer's search; but, alas! artifice could not obtain impunity even to reputation; the artery remained bedded in fat, and untouched; the nerve alone was separated from the mass, and bore the mark of the ligature.

May the relation of these disgraceful occurrences, selected from numerous similar ones, which, to my knowledge, have taken place both in London and in Edinburgh, but which I am unwilling to relate, serve as a proof of the danger arising from ignorance of the principle I have just proposed.

### Division IX.

### *Of the Necessity of such Methods in the present State of Europe.*

While such important operations remain in so vague a state, what ease would not such measurements confer; when, in the cockpit of the man of war, or on the field of battle, the perplexed and bewildered surgeon knows not where to turn him, but must leave an hundred operations in an hour to an inexperienced assistant.

At a time when a successful conspiracy of adventurous ruffians are, at the point of the bayonet, establishing, from the Don to the Douro, from the Scheldt to the Catarro, over all the fairest provinces of Europe, the most dreadful of military despotisms;—at a time when, to the shout,

"Avance!

"Avance! Avance!" louder and more tremendous than the noise of their guns, the myriad legions of France are carrying death and devastation before them, and sweeping man and civilization from the face of the continent; — at a time when he who has "sceptres tipped with coronets for his pallisadoes, kingdoms for his Martello towers, and kings for his centinels," threats at once the liberties and the existence of Britain, and stains every step of his career with perfidy and blood; — at a time when it is the duty, it is the wish of every man of honour, successfully to defend, or, covered with wounds, nobly to die for the liberties of Britain and the emancipation of Europe; when all this is the case, what are not his duties, whose sole profession it is, aided by science, to rescue from death or agony those whom tyranny has attempted to destroy; the genius of whose godlike art is to save both friends and foes, and upon whose knowledge the existence and the fate of armies may in no small degree be said to depend.

Never were the duties of the Military Surgeon more complex or more formidable; never did he stand in need of greater precision; and in his art, at least, he ought to recollect that no commissions are sold, the precisest knowledge alone entitles to, infallibly secures, pre-eminence and fortune.

